# **Issue Competition and the Social Construction of Target Populations: Alternative Suggestions for the Study of the Influence of Populist Radical Right Parties on Health Policy and Health Outcomes** Comment on "A Scoping Review of Populist Radical Right Parties’ Influence on Welfare Policy and its Implications for Population Health in Europe"

**DOI:** 10.34172/ijhpm.2020.157

**Published:** 2020-08-22

**Authors:** Carole Clavier, Elisabeth Martin, France Gagnon

**Affiliations:** ^1^Departement of Political Science, Université du Québec à Montréal, Montréal, QC, Canada.; ^2^Faculty of Nursing, Université Laval, Québec, QC, Canada.; ^3^School of Administration Sciences, Université TÉLUQ, Québec, QC, Canada.

**Keywords:** Political Parties, Issue Competition, Target Populations, Policy Feedback, Health Effects

## Abstract

Rinaldi and Bekker ask whether populist radical right (PRR) parties have an influence on population health and health equity. The assumption is that this influence is negative, but mediated by political system characteristics. Starting from the authors’ premise that the positions of PRR parties on welfare policies are a good proxy for health outcomes, we build on political science literature to suggest further avenues for research. The equivocal relationship between political parties and the ownership of specific healthcare, health insurance and public health issues invites studies that break down party positions relating to different health policy issues. As policy-makers use social representations of target populations to make policy decisions and anticipate the feedback these decisions might generate, it is worth studying how PRR parties influence societal, institutional and partisan perceptions of deserving and undeserving populations, even when they are not in government

Rinaldi and Bekker’s scoping review of the literature asks whether populist radical right (PRR) parties have an influence on population health and health equity.^1^ This is a classic political science question: do parties matter? And how? Do their positions represent public opinion? In this commentary, we lay out some of the key conceptual issues underpinning the literature on the influence of political parties on public policies so as to suggest further research avenues for the practical study of how political parties influence health. Taking stock of the ambiguity of the literature on partisan influence on public policy, we start by questioning Rinaldi and Bekker’s premise that welfare policies are a good proxy for health outcomes. This leads us into exploring the literature on issue competition and its relevance to health policy and outcomes. Which political party owns the issue of health policy varies according to the dimension of health under consideration and according to institutions and context. Consequently, empirical and comparative studies of PRR parties’ positions on diverse aspects of health policy would help better understand their influence on health policy and health outcomes. Finally, we question how political parties matter for policy decisions, even when they are not in government. Here, the literature points to the importance of how society and, ultimately, policy-makers perceive different categories of publics or target populations for their policies. Another avenue for studying the influence of PRR parties on health policy and health outcomes, therefore, would be to question the pathways of their influence on the framing of certain populations as deserving of public subsidies and services and on the framing of other populations as undeserving of public subsidies and services.

## Welfare Policies, Health Outcomes and the Influence of Political Parties on Public Policies

The impact of political parties on public policy is disputed: while Rose^2^ argued that the importance of political parties was limited compared to societal and institutional factors, studies generally indicate a positive correlation between the party in government and levels of public expenditure. Left wing parties are supposed to spend more than right wing parties, who are assumed to be more aligned with neoliberal macroeconomic policy principles.^3^ But even these conclusions are mediated by the types of political regimes and institutions, the configurations of power between parties in government or the types of issues under consideration.^4^ In their review, Rinaldi and Bekker take stock of this literature by including studies that explore different pathways for the influence of PRR studies, including the broader influence of ideology, and by exploring the impact of institutions on this influence. However, the literature on partisanship and public policy raises questions regarding the authors’ key premise that the positions of PRR parties on welfare policies are a good proxy for health outcomes. We do not dispute that welfare policies do influence health outcomes, nor that there is little literature specifically questioning the effects of PRR parties on health outcomes. This said, this premise is worth unpacking as its terms refer to complex realities, suggesting different directions for further research on the influence of PRR parties – and parties in general – on health policy and health outcomes.

First, the reference to health outcomes is ambiguous in the paper. Does it refer to health results at the population level or to health inequalities among groups of populations? Welfare policies may have different impacts on different groups of population or in different areas, and thus have very different outcomes in terms of health inequity. This echoes the inverse care law, which posits that those with the greatest healthcare needs are less likely to use health services. It leads deprived populations to receive less care and experience reduced access in comparison to more affluent populations, even in universal healthcare systems,^5^ due to services organisation and other factors. Thus, studying the influence of PRR party ideology on policy implementation might be a worthwhile research avenue.

Second, the article considers only the influence of PRR parties on welfare policies but, given the breadth of the social and political determinants of health, PRR parties may influence health outcomes through policies other than healthcare and welfare. For instance, their influence on fiscal policy may have consequences for the structure of inequalities in society, which in turn may have consequences for health outcomes.^6^

Third, what counts as “welfare policies” is a bit ambiguous in the paper: do policies redistributing wealth “through progressive taxation and/or social benefits and provision”^1,2^ include healthcare policies and public health policies? Or some aspects of these policies only? This has implications for the study of the influence of PRR parties on health outcomes as they may have different influences on public health policies according to the context and to existing institutions. It is especially on this statement that we wish to insist in this commentary. As mentioned above, the literature about the influence of political parties on public policies is equivocal in general. It is also equivocal when it comes to the relationship between public health policy and partisan politics. This suggests avenues for further research.

## Issue Competition and the Multiple Dimensions of Health Policy

Political sociology addresses the “do parties matter” question by focusing on the capacity of political parties to reframe public problems in their own terms (competition for the control of the agenda) and on the capacity of political parties to determine which public problems will make it onto the agenda (issue competition).^7^ The latter means that some parties are strongly associated with specific policy issues. Who, then, owns the issues of the welfare state and health policy? The literature on issue competition generally concludes that social democratic parties own the issue of the welfare state. A recent review confirmed these findings, concluding that “social democratic parties have been the main agents of welfare state expansion and egalitarian social policy and the opposition to retrenchment after the 1980s.”^8,9^ However, social democratic parties have also introduced considerable welfare state reforms under the influence of neo-liberalism and new public management since the 1990s. Their status as guardians of the welfare state allowed them to introduce reforms that other parties might not have had the leeway to introduce.^9,10^ Other reviews on party politics and the welfare state even conclude that left-wing and right-wing parties do not necessarily hold very different views on welfare policy. According to Häusermann et al,^11^ the distinction between traditional party positions may have faded under the influence of electoral reforms, institutions and interactions with the electorate.

The evidence regarding the ownership of the health issue among political parties is even more mixed than that regarding the welfare state. It is highly dependent on context, on institutions and on which aspect of health policy one focuses on. For instance, the preference for public or private health insurance is strongly associated with, respectively, left-wing and right-wing political parties, mediated by the institutions of the political system. Third-parties defending the cause of public health insurance had more institutional opportunities to play a role in Canadian politics than in the United-States, thus leading to two very distinct health insurance policies in the two countries.^12^ But the evidence regarding prevention and health promotion policies is not so clear-cut. In Europe, Mackenbach and McKee^13^ have found a long-term trend associating social-democratic governments and some areas of health-related prevention (smoking prevention). But they also conclude that “social-democratic government in recent decades has not been very conducive to health policy.”^13^ In some cases, prevention and health promotion policies have been fostered as part of reforms to help curb healthcare expenditure. In Canadian provinces, the social investment paradigm has contributed to the adoption of health promotion programmes because these were expected to reduce public spending on healthcare by improving the general health of the population.^14^

 In sum, many parties have different perceptions of healthcare and public health policy. PRR parties may take actions that could have positive effects on health in some areas of public health policy, while social democratic parties may weaken some aspects of public health policy. In other words, the effects of PRR parties on public health policy are potentially more varied, and equivocal, than their effect on welfare policy strictly speaking. The implications of these ambiguous findings warrant further research on the positions that PRR parties take regarding different aspects of healthcare, health insurance or public health policy, and on the pathways for their influence on health policy and health outcomes.

## How Political Parties Influence Policy-Makers’ and Society’s Perceptions of Deserving and Undeserving Populations

This segues into our second suggestion for further research on the influence of PRR parties on health outcomes, namely to question the pathways for political parties’ influence, even when they are not in government. They may influence what governments decide to do or not to do^15^ by framing issues in a certain way or by promoting certain policy issues. This is the direction that Rinaldi and Bekker’s paper points to when they highlight the importance of the indirect effects of welfare chauvinism. Discussing how PRR parties have influenced the perception of who is deserving and who is undeserving of social benefits, they explain how mainstream political parties in Denmark and Sweden have absorbed PRR parties’ representations of deserving and undeserving publics, thus implementing welfare reforms restricting access to benefits for immigrant or minority populations.^1^ This echoes Schneider and Ingram’s^16^ theory of the social construction of target populations and of the role of policy-makers’ anticipatory feedbacks. As Schneider and Ingram explain, the prevalent representations of certain categories of populations in a society influence what they “should” get from public policies. Groups perceived as not so powerful but deserving are more likely to get benefits from public policies than groups perceived as weak and undeserving. Similarly, healthcare systems are built on norms and values that differentiate between deserving and undeserving populations: the extent and access to services vary depending on the healthcare systems’ founding postures regarding equality, liberty or individual responsibility.^17^ This may also feed into policy-makers’ perceptions of deserving and undeserving populations. Policy-makers then anticipate how the general public (ie, the electorate) will perceive how they allocate public funds and adapt their strategies accordingly. Policy-makers allocating benefits to powerful groups deemed undeserving of public support are likely to do so in a hidden manner, so that disapproval from the general public does not harm their reputation. Alternatively, policy-makers may reject or limit benefits granted to groups considered as weak and undeserving, because they anticipate positive feedback from the general public and, thus, a better chance at re-election. Combined with the results of Rinaldi and Bekker’s review, the theory of social construction of target populations and policy-makers’ anticipatory feedback invites further research into how parties influence societal and policy-makers’ construction of deserving and undeserving populations. How do parties shape the representations of different population groups? How do parties interact with citizens and other societal actors in forming these representations? Political parties may influence health outcomes directly through their policy and instrument choices when they are in government. They may also influence health outcomes indirectly even when they are not in government by shaping the representations of who is deserving of public benefits or not. The mechanisms through which they exert this influence among the general population, the media, social networks, other political parties and public officials are worth studying further.

## Ethical issues

 Not applicable.

## Competing interests

 Authors declare that they have no competing interests.

## Authors’ contributions

 CC drafted the paper. EM and FG revised and completed the paper. All authors approved the final version.

## Authors’ affiliations

^1^Departement of Political Science, Université du Québec à Montréal, Montréal, QC, Canada. ^2^Faculty of Nursing, Université Laval, Québec, QC, Canada. ^3^School of Administration Sciences, Université TÉLUQ, Québec, QC, Canada.
